# Sporadic renal hybrid oncocytic/chromophobe tumor in a young man: A case report

**DOI:** 10.1097/MD.0000000000016641

**Published:** 2019-08-16

**Authors:** Bingbing Hou, Chaozhao Liang

**Affiliations:** a Department of Urology, the Fourth Affiliated Hospital of Anhui Medical University; b Department of Urology, the First Affiliated Hospital of Anhui Medical University, Hefei, China.

**Keywords:** chromophobe, hybrid tumor, oncocytoma, renal cell carcinoma

## Abstract

**Rationale:**

Hybrid oncocytic/chromophobe tumor (HOCT) is defined as tumor composed of renal oncocytoma (RO) and chromophobe renal cell carcinoma (CHRCC). Sporadic HOCT is extremely rare, the preoperative diagnosis is difficult, and no guidelines for clinical therapy. We report a case who is the youngest male patient of sporadic HOCT in the world, review the previously reported cases, and share the clinical features, diagnosis, treatment, and prognosis of HOCT.

**Patient concerns:**

A 30-year-old man was admitted with the complaints of incidental right renal tumor detected by abdominal ultrasound. He had no complaints of urological symptoms, abdominal pain, osphyalgia, and hematuria. Abdominal contrast-enhanced computed tomography revealed an 85 mm × 80 mm × 80 mm unilateral and solid renal mass, and no findings of metastases.

**Diagnosis:**

The preoperative diagnosis was right renal tumor.

**Interventions:**

Laparoscopic right radical nephrectomy was performed.

**Outcomes:**

Histopathology demonstrated a mixture of cells with the morphologic features of those seen in CHRCC and RO. The patient was final diagnosed as sporadic HOCT. After follow-up of 14 months, the patient had no complaints and evidence of disease recurrence.

**Lessons:**

Sporadic HOCT is extremely rare. It is possible that core biopsy could improve diagnostic accuracy. Laparoscopic radical nephrectomy or nephron sparing surgery should be considered the clinical therapy of the sporadic HOCT patients. The clinical behavior of HOCT is still entirely uncertain and should be proved by studies with available long follow-up.

## 1 Introduction

Renal oncocytoma (RO) is a benign neoplasia and chromophobe renal cell carcinoma (CHRCC) is a malignant tumor. Hybrid oncocytic/chromophobe tumor (HOCT) is defined as tumor composed of varying amounts of cells with features of RO and CHRCC. Usually, the numerous HOCT have been initially described in patients with Birt–Hogg–Dubé syndrome (BHDS) or with renal oncocytosis without BHDS.^[1]^ Sporadic HOCT, which means HOCT occurs in patients without BHDS or renal oncocytosis, is extremely rare. We present a case of an unusual renal cell carcinoma: sporadic HOCT in a young man. The aim of the present study was to share the clinical features, diagnosis, treatment, and prognosis of HOCT.

## 2 Consent

Informed consent was signed by the patient for the publication of this report and related images.

## 3 Case report

**Figure 1:**
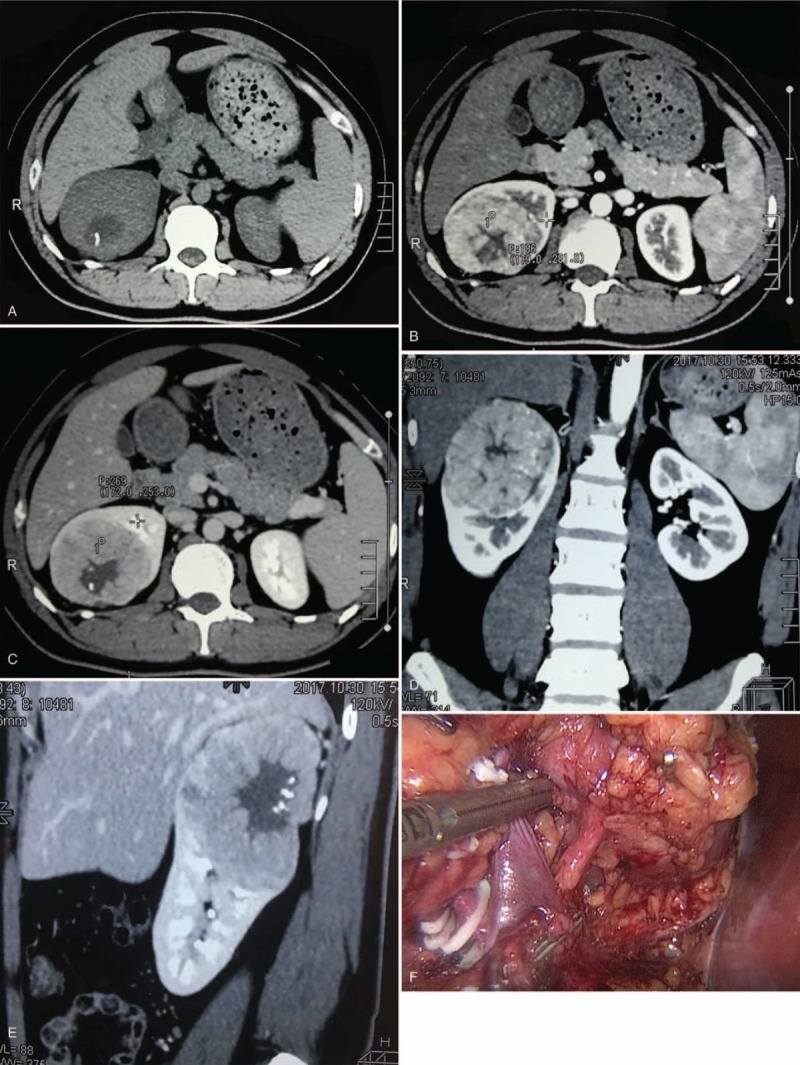
Preoperative CT and intraoperative imaging. A, Plain scan shows a large mass measured 8.5 cm in diameter in the upper pole of the right kidney, displaying central stellate hypodensity areas inside with punctate calcification. B, The mass has an apparently enhancement in arterial phase. C, The mass shows a relatively low density compared with parenchyma in venous phase. D, The mass in contour view. E, The mass in sagittal view. F, Laparoscopic right radical nephrectomy was performed and the mass is found to have been supplied by independent artery. CT = computed tomography.

**Figure 2:**
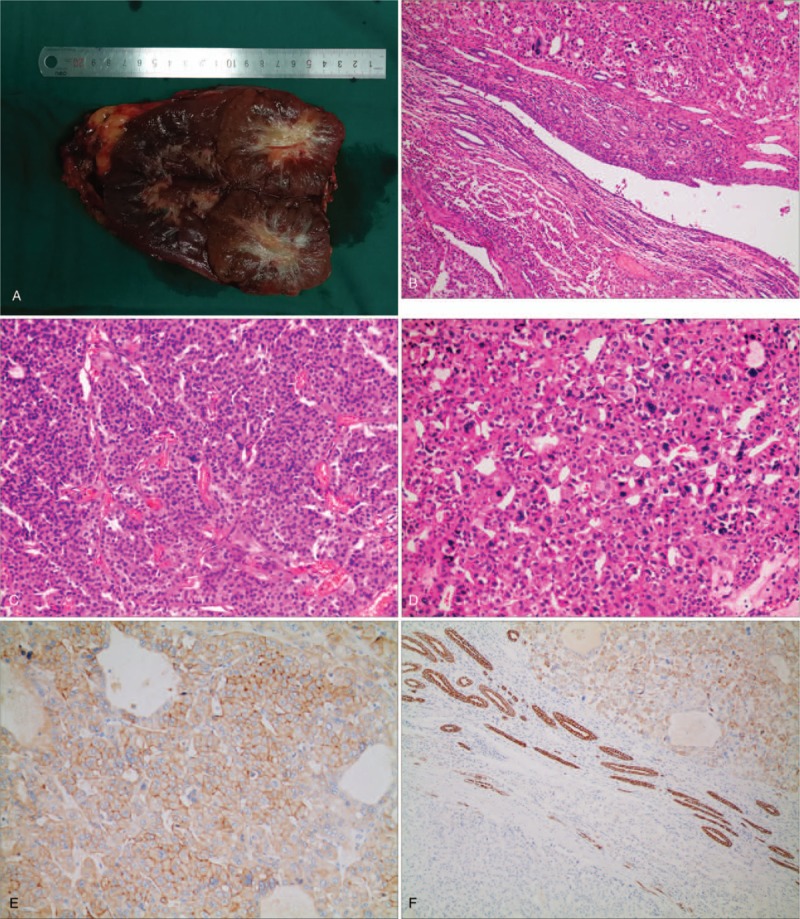
Macro- and microscopic findings of surgical specimens. A, The tumor was macroscopically well marginated and dark pink-brownish in color, fibrotic strands resembling central scar were seen. B, Microscopic findings with HE staining reveal the tumor is composed of CHRCC and RO (×100). C, HE staining of RO (×200). D, HE staining of CHRCC (×200). E, Immunohistochemical staining with CD117 is diffusely positive on the cell membrane (×200). F, CK7 showed diffuse granular cytoplasmic staining of chromophobe-like cells, whereas oncocytic cells showed absent staining (×100).

A 30-year-old man was admitted with the complaints of incidental right renal tumor detected by abdominal ultrasound. His temperature and blood pressure was normal. He had no complaints of urological symptoms, abdominal pain, osphyalgia, and hematuria. No cutaneous lesion or history of spontaneous pneumothorax was noted. All blood and urine examinations were within normal limits. Abdominal contrast-enhanced computed tomography revealed a heterogenous mass of 85 mm in diameter in the upper pole of the right kidney, displaying central stellate hypodensity areas inside with punctate calcification, and no findings of metastases (Fig. 1A–E). Laparoscopic right radical nephrectomy was performed under a preliminary diagnosis of right renal tumor. Intraoperative found the tumor is supplied by independent artery (Fig. 1F). Grossly, the tumor size was 85 mm × 80 mm × 80 mm, rounded, well-defined, and dark pink-brownish. The fibrotic strands resembling central scar were seen in the tumor (Fig. 2A). The pathology of the tumor is composed of neoplastic cells predominantly arranged in a solid-alveolar pattern, with nuclei showing mild nuclear pleomorphism and abundant granular eosinophilic to oncocytic cytoplasm (Fig. 2B–D). Tumor is positive immunostaining with PAX8, SDHB, CD117. CK7 showed diffuse granular cytoplasmic staining of chromophobe-like cells, whereas oncocytic cells showed absent staining (Fig. 2E and F). Negative results were seen for CA9, HMB45, P504S, TFE3, Vimentin. We examined cytogenetic abnormalities of the tumor. No known or suspected pathogenic mutations were found in the 21 genes of genetic tumor, including VHL, MET, and FLCN genes etc. There is no evidence of BHDS or renal oncocytosis and the patient was final diagnosed as sporadic HOCT. The clinicopathological stage is pT2bN0M0. After follow-up of 14 months, the patient had no complaints and evidence of disease recurrence.

## 4 Discussion

RO in youth is an infrequent renal neoplasm and the one with the CHRCC component is extremely rare. HOCT have been described in patients with BHDS or with renal oncocytosis.^[1]^ Sporadic HOCT was reported later by a few authors reviewing their series of RO, CHRCC, and misclassified eosinophilic renal carcinoma. The real morbidity of sporadic HOCT is not known because of its possible poor recognition. Petersson et al^[2]^ reported that sporadic HOCT cases represented only about 2% (14/749) of previously diagnosed RO and CHRCC without evidence of BHDS or renal oncocytosis, as observed in another HOCT series.^[3]^ Sporadic HOCT often occur in adult patients with no sex predilection and an age ranged from 40 to 79 years (median 63.5 years). The size of tumors ranged from 2 to 11 cm (mean 5.2 cm).^[2]^ Our case is an only 30-year-old man, who is the youngest male patient of sporadic HOCT in the world, verify through reviewing of previous reported cases as much as possible.^[2,3]^

The preoperative diagnosis of HOCT is very difficult. Clinically, sporadic HOCT is mostly unilateral and solitary, whereas HOCT in individuals with BHDS or oncocytosis are often bilateral and multiple. Patients with BHDS usually have fibrofolliculom, pulmonary lesions including bullae and spontaneous pneumothorax.^[4]^ But no specific clinical symptoms are reported in patients with sporadic or associated with renal oncocytosis. Bhatnagar et al^[5]^ reported 9 patients with pathologically confirmed as HOCT had available preoperative contrast-enhanced CT examinations, they found HOCT on CT to have 2 distinct patterns—a heterogenous enhancement pattern and an “oncocytoma-like” pattern with a central stellate hypodensity. However, radiographic techniques may not be a reliable method to make an accurate prospective diagnosis of HOCT for its similarity features with other renal tumors, particularly RO.

HOCT possess the characteristics of CHRCC and RO simultaneously. In renal tumor biopsy, HOCT can only be found incidentally. It was reported that about 15% of RO diagnosed by renal tumor biopsy were actually HOCT, which raise the issue of a potential misleading diagnosis when HOCT is biopsied.^[6]^ As well, someone who diagnosed as RO should consider the possibility of HOCT. HOCT with BHDS or oncocytosis and sporadic one share similar morphologic features and show differences at the cytological Level. HOCT associated with BHDS is associated with mutations in the FLCN gene.^[7]^ Sporadic forms may present with numerous molecular anomalies of chromosomes, and lack of mutations in FLCN gene.^[2]^ We examined 21 genes of genetic tumor, including VHL, MET, and FLCN genes, etc. No known or suspected pathogenic mutations were found. These findings further confirm the diagnosis of Sporadic HOCT.

There are no guidelines for clinical therapy of the sporadic HOCT patients, because the researches for this tumor were mostly retrospective with relatively small sample size. The majority of urologists and pathologists^[2,3]^ reported that they recognize HOCT, as a low malignant potential tumor managed similar to renal tumors of other type, should be first considered the clinical therapy by nephron-sparing surgery. If partial nephrectomy is not technically suitable, onetime radical nephrectomy should be performed. The sporadic HOCT usually pursue a favorable clinical course, as no recurrence and metastasis has been documented.^[2,8,9]^ However, the follow-up should be no longer than 10 years.^[10]^ Therefore, as a low malignant potential may not be eliminated, the prognosis of HOCT can only be found out with longer follow-up. In our case, it was not associated with lymph node or distant metastasis after a 14-month follow-up.

## 5 Conclusion

Sporadic HOCT is an extremely rare tumor. The preoperative diagnosis is difficult and easily misdiagnosed as RO. It is possible that core biopsy could improve diagnostic accuracy. Laparoscopic radical nephrectomy or nephron sparing surgery should be considered the clinical therapy of the sporadic HOCT patients. The clinical behavior of HOCT is still entirely uncertain and should be proved by studies with available long follow-up. Urologist and pathologist must pay more attention to this rare tumor.

## Author contributions

**Supervision:** Chaozhao Liang.

**Writing – original draft:** Bingbing Hou.
